# Science-based health innovation in Tanzania: bednets and a base for invention

**DOI:** 10.1186/1472-698X-10-S1-S4

**Published:** 2010-12-13

**Authors:** Ronak Shah, Peter A Singer, Abdallah S Daar

**Affiliations:** 1 McLaughlin-Rotman Centre for Global Health, at the University Health Network and University of Toronto, MaRS Centre, South Tower, Suite 406, 101 College Street, Toronto, Ontario, M5G 1L7, Canada

## Abstract

**Background:**

Tanzania is East Africa’s largest country. Although it is socially diverse, it has experienced general political stability since independence in 1964. Despite gradual economic development and Tanzania’s status as one of the biggest recipients of aid in Africa, health status remains poor. This paper explores Tanzania’s science-based health innovation system, and highlights areas which can be strengthened.

**Methods:**

Qualitative case study research methodology was used. Data were collected through reviews of academic literature and policy documents, and through open-ended, face-to-face interviews with 52 people from across the science-based health innovation system over two visits to Tanzania from July to October 2007.

**Results and discussion:**

Tanzania has a rich but complex S&T governance landscape, with the public sector driving the innovation agenda through a series of different bodies which are not well-coordinated. It has some of the leading health research on the continent at the University of Dar es Salaam, Muhimbili University of Health and Applied Sciences, the National Institute for Medical Research and the Ifakara Medical Institute, with strong donor support. Tanzania has found developing an entrepreneurial culture difficult; nevertheless projects such as the clusters initiative at the University of Dar es Salaam are encouraging low-tech innovation and overcoming knowledge-sharing barriers. In the private sector, one generics company has developed a South-South collaboration to enable technology transfer and hence the local production of anti-retrovirals. Local textile company A to Z Textiles is now manufacturing 30 million insecticide impregnated bednets a year.

**Conclusions:**

To have a coherent vision for innovation, Tanzania may wish to address some key issues: coordination across stakeholders involved with health research, increasing graduates in health-related disciplines, and building capabilities in biological testing, preclinical testing, formulation and standardization, and related areas important to moving from basic research to applications. The private sector can be encouraged to innovate through improved access to financing, and incentives for R&D. The diaspora community represents an untapped source for partnerships and access to other developing world markets and technology. The government may wish to set up mechanisms to encourage south-south collaborations, and to bring the public and private sector together around specific projects to help realize the country’s innovation potential.

## Background

Located on the Indian Ocean, Tanzania is East Africa’s largest country with a population of 41.5 million [1]. Although it is socially diverse, with some 125 ethnic groups, it has experienced uninterrupted peace and general political stability since its independence in 1964. With a historical legacy of socialist principles, Tanzania has gradually undertaken economic reforms that have increased private sector activity and opened up the economy to global competition. Reforms have included privatizations of state firms, on-going improvements to Tanzania's weak infrastructure, increased access to information and communication technologies including Internet broadband access through fiber-optic cable, and increasing private sector activity in tourism, retail, mining, transport, communication and agriculture sectors. As a result, Tanzania’s GDP has grown at a rate of more than 5% over the last five years, reaching 7.5% in 2007 [2].

Despite these statistics Tanzania remains one of the poorest countries in the world, with the majority of citizens dependent on agriculture and engaged in small and medium sized enterprises [3]. It is positioned 152^nd^ on the UNDP Human Development Index, with a per capita GDP of $US 430 [1]. Tanzania is among the largest recipients of aid in Africa, with over 34% of the national budget in the 2008/2009 financial year financed through aid.

With annual per capita spending of $22 USD per person on health, average life expectancy is 53 years [4]. Communicable diseases account for the majority of Tanzania’s disease burden. HIV/AIDS is the leading cause of mortality in the country, followed by malaria. Tuberculosis, diarrheal diseases, and lower respiratory infections are other common diseases. Non-communicable diseases such as cardiovascular disease and diabetes still account for a small proportion of the disease burden but are on the rise [4,5].

Science and technology (S&T) have long been important in Tanzania. In 1968, it established a national research council (Tanzania National Science Research Council), one of the first African countries to do so. Its first S&T policy was formulated in 1986 to determine strategies for the application of science and technology development, with a focus on agriculture and livestock. In 1990 the Ministry of Science, Technology and Higher Education was established and made responsible for S&T policy. Tanzania has developed an extensive S&T education system, including 44 higher education institutions that offer S&T training and undertake research.

In this paper we present research on science-based health innovation in Tanzania, including biotechnology. By science-based health innovation, we mean technological innovation across a spectrum of sophistication, from vaccines, pharmaceuticals, and medical devices to some plant medicines where attempts to scientifically standardize or characterize medicines have been made. We take a broad definition of innovation as not only new-to-the-world innovation, but also the diffusion, adaptation and use of technologies. We use the OECD definition of biotechnology: ‘the application of science and technology to living organisms, as well as the parts, products and models thereof, to alter living or non-living materials for the production of knowledge, goods and services’ [6].

The purpose of this paper is to describe and analyze science-based health innovation using an innovation system framework, which takes into account the wide variety of stakeholders who contribute to the innovation process and emphasizes the dynamic interaction and knowledge flow between them [7]. The study was undertaken at the invitation of the then Minister of Science, Technology and Higher Education, who later became the Minister of Communications, Science and Technology. It is the first study of its kind on Tanzania with an emphasis on commercialization and understanding how knowledge translation happens in the area of health product development.

## Methods

A case study research methodology was used in this study [8]. Data was collected through reviews of academic literature and policy documents and through open-ended, face-to-face interviews in Tanzania. Interviewees were identified through snowball and purposive sampling. We interviewed 52 people from across the science-based health innovation system, including government officials (n=16), researchers (n=22), entrepreneurs (n=6), international donors (n=6) and non-governmental organization representatives (n=2), over two visits to Tanzania from July to October 2007. Since our initial research visits, we have continued to engage stakeholders to address key challenges identified in this case study.

All quotes are from the interviews unless noted, and with permission. This study was approved by the Office of Research Ethics of the University of Toronto.

## Results and discussion

In the following sections, we describe and discuss Tanzania’s science-based health innovation system.

### Government

In February 2008 the Ministry of Communication, Science and Technology (MCST) was created with a mandate to further develop policies and guidelines and co-ordinate science, technology and innovation in Tanzania. S&T policies are being revised to include not only S&T but also innovation. These reforms are being initiated in support of the government’s Vision 2025 objective of ‘transform[ing] the economy into a strong, resilient and competitive one, buttressed by science and technology’ [9]. In the words of Minister Peter Msolla, the Tanzanian Minister of Communications, Science and Technology, to fellow ministers meeting for the High-level Segment of the UN Economic and Social Commission’s meeting on 1 July 2008, ‘without a dose of innovation [in Tanzania], the macro-economic gains achieved over the years through the implementation of sound economic policies would be wiped out.’

Another important body in S&T development is the Commission for Science and Technology (COSTECH), a semi-autonomous institution under the MCST responsible for coordination and promotion of research in Tanzania since 1986. COSTECH advises the government on S&T, and it has eight sectoral advisory committees including: Agriculture and Livestock, Social Sciences, Basic Sciences, Natural Resources, Environment, Medicine and Health, Industry and Energy. Biotechnology is one area of interest under basic sciences, with an emphasis on agricultural biotechnology.

The Ministry of Health and Social Welfare is mandated to undertake research, policy, coordination and regulation of health in the country. An important stated focus of health research in the country is related to traditional medicine. The government is developing ways to integrate traditional medicine into mainstream medicine, recognizing that at least 70% of Tanzanians use traditional medicine as the first port-of-call for primary care – particularly those in rural areas. The Traditional Medicines and Alternative Medicines Act guides the use and promotion of traditional medicine in Tanzania. A Traditional Medicines Board under the Ministry of Health and Social Welfare has been set up to regulate traditional medicines in the country.

The Medical Stores Department, a parastatal agency under the Ministry, is the main procurer, supplier and distributor of essential medicines for the public sector, and is the primary supplier to faith based organizations and non commercial groups providing health services in Tanzania. The Pharmaceutical Services Unit of the Ministry has been set up to guide the development of the local pharmaceutical sector and to ensure the Medical Stores Department performs according to the MSC Act (1993). The National Medicine Policy (NMP, 1991) and Pharmaceutical Master Plan (1992-2002) are being reviewed to reflect a shift from the push to pull supply system and increase the promotion of the local manufacturing sector. A critical barrier we observed was around shortage of staff and operational capacity within the Pharmaceutical Services Unit, along with a shortfall of trained pharmacists in the country.

The government through COSTECH established in 2002 a multi-sectoral National Biotechnology Advisory Committee (NBAC) with the mandate of advising the government on all issues pertaining to the safe development and application of biotechnology. A National Biotechnology Policy has been drafted. The main objective of the policy is to ensure that Tanzania has the capacity and capability to capture benefits arising from health, agricultural, industrial and environmental applications of biotechnology, while protecting and sustaining the safety of the community and the environment. Specific policy objectives include the development of a coordinated biotechnology strategy; establishing bio-safety regulations and guidelines; conserving genetic resources; linking R&D and industrial capacity for biotechnology in Tanzania; and a number of innovation-related aims such as strengthening innovative financing and intellectual property (IP) rights.

Despite the country’s significant S&T infrastructure in both health and biotechnology, there has been a relative lack of governmental investment in both research infrastructure and investment in research to support the type of innovation envisioned in governmental policies. It has been estimated that about 0.18% of GDP is invested in research and development (R&D) [10]. However, in 2009 the President of Tanzania announced that the government would increase its funding for R&D to one per cent of GDP for the financial year 2009/2010, amounting to 311 billion Tanzanian shillings (approximately US$235 million). A recent survey of funds flowing to R&D institutions conducted by COSTECH indicate that the donor contribution to R&D expenditure is the largest at 52%, followed by government at 34%, and a small proportion from institutions’ internally generated funds at 14% [11]. Currently there is no significant government funding dedicated towards commercialization of innovation, such as proof of concept funds or product development funds.

The Tanzania Food and Drug Authority (TFDA) established under the Food, Drugs, and Cosmetics Act (2003) is the country’s key regulatory body responsible for controlling the quality, safety and effectiveness of food, drugs and medical devices. It is a semi- autonomous agency under the Ministry of Health and Social Welfare, with 50 drug inspectors and an emphasis on a science-based approach. With regards to regulation and registration of clinical trials locally, there seems to be jurisdictional overlap between the National Institute for Medical Research’s (NIMR’s) National Health Research Ethics Committee and TFDA. They are working together to address this issue by forming a joint ethics approval process. We found that the TFDA is unique as a regulator, as it also plays a role in the promotion of the local pharmaceutical manufacturing sector. Having realised the need to develop the local pharmaceutical industry, it developed a concept paper, which it presented it to the Minister of Health. As Mrs M.N. Sigmondo, the director general of the TFDA, explains: “The Ministry approved the concept paper, and now we have in place a sort of plan covering ten years – 2006 to 2016 – that tries to elaborate how best as a country we could utilize the existing potential in the country to promote local production, including research and development.”

The Business Registration and Licensing Agency (BRELA) under the Ministry of Industry, Trade and Marketing is mandated to regulate intellectual property rights in Tanzania (except copyrights which are under the Copyright Society of Tanzania). Most locally owned patents are in the fields of engineering and agriculture, with none identified in health biotechnology. Tanzania has been a member of the World Trade Organization since 1995 and has thus ratified the Trade Related Intellectual Property Rights (TRIPS) agreement. Due to its classification as a Least Developed Country, it is exempt from implementing, enforcing or applying TRIPS provisions on patents with respect to pharmaceutical products until 1^st^ January 2016. However, Tanzania has so far not made much use of this exemption. The Patent Act (1987) makes patents available for both processes and products without expressly excluding pharmaceutical products. The Act also does not appear to have provisions for data protection and exclusivity. According to the Patent Act (1987) Section 37 (2), parallel imports from abroad are not admitted, and, though compulsory licensing is permitted, there are restrictions. These stipulations have be critiqued elsewhere as limiting Tanzania’s capacity to produce essential medicines [12,13]. The government has initiated the process of amending the Patents Act to revise and consolidate the entire intellectual property system including other laws such as Trade and Service Marks Act, 1986.

We found two critical challenges with respect to intellectual property protection. First is the lack of awareness around the need for intellectual property protection amongst the research community. COSTECH in association with BRELA and the World Intellectual Property Organization have established a new unit housed within COSTECH to address this gap. The Tanzania Intellectual Property Advisory Service has been set up to increase awareness and support around intellectual property amongst the science community locally, though the extent to which this has had an impact is not yet clear. Second is the weak system of policing patents. We came across scientists and companies who were reluctant to patent their research, as it requires disclosing confidential information and there is no strong system to enforce the patents should they be violated. This naturally has impacts on knowledge translation.

### Research institutes and universities

There are a total of 44 higher education institutions in Tanzania. Health and biotechnology research is primarily conducted by three categories of institutions: governmental health research institutes, universities (public and private), and private health research institutes. In 2006/07 total student enrolment in both private and public universities and university colleges and institutes was 49,967, with public universities enrolling a total of 39,218 students or 78.4% of the total students [14]. Despite the overall increase in enrolment numbers in tertiary education, Tanzania’s numerous strong research institutes and universities have been struggling with recruiting students in science and technology including biotechnology. This is partially due to lack of new research infrastructure and trained staff.

#### National Institute for Medical Research

The National Institute for Medical Research (NIMR) plays a critical role in medical research in Tanzania. NIMR was set up as a parastatal organization under the Ministry of Health and Social Welfare in 1979, and is responsible for undertaking and coordinating health research in Tanzania. It is headquartered in Dar es Salaam but has ten research stations and centers throughout the country. Its current research priority areas are malaria, filariasis, trypanosomiasis, onchocerciasis, schistosomiasis and sexually transmitted diseases including HIV/ AIDS.

NIMR has around 87 full-time research scientists and 34 technical staff. NIMR collaborates significantly with other Tanzanian research organizations such as Hurbert Kairuki Memorial University (Dar Es Salaam), and international groups such as the National Institutes of Health (US) and University of Toronto (Canada). They have also set up a collaborative biotechnology laboratory at the Kilimanjaro Christian Medical Centre in Moshi which conducts multidisciplinary research on prevention, control and treatment of the three poverty related diseases HIV/ AIDS, malaria and tuberculosis.

We found scientists at NIMR who had developed an innovative extraction process for the active ingredient of *Artemisia annua*, a plant which forms the basis of artemisinin compounds for the treatment of malaria and which grows in abundance in Tanzania. However, the process had never been commercially explored, partly due to the reluctance of scientists to share the information outside the institution because of the lack of IP protection. Currently, therefore, all Tanzanian *Artemisia* is farmed, dried and exported to Kenya, where it is extracted by a Kenyan private sector company, before being shipped to Switzerland where it is further processed for use in the anti-malarial Coartem ® produced by Novartis and later shipped back to Tanzania to be sold. Little commercial value is captured locally.

#### University of Dar es Salaam

The University of Dar es Salaam (UDSM) is the oldest and largest university in Tanzania. In 2008, UDSM had 5775 undergraduate students and 2552 graduate students. The university has a biotechnology unit within its Faculty of Science, where annually around 35 students are trained at the undergraduate level and 10 students at the graduate level. Health research includes the identification of antimicrobial agents from Tanzania’s biodiversity.

Outside the biotechnology unit, there are a number of research groups in the area of natural products and traditional medicine. For example Prof. Nkunya, a Professor of Chemistry at UDSM, runs a research group focused on medicinal chemistry and traditional medicine and has established linkages with GIBEX, an international consortium on biotechnology research. They are focusing on biodiversity, bio-exploration and natural product research including identifying wild mushrooms as a source of biomedical products. With respect to commercialization of research, the University has developed a technology transfer policy and a technology transfer office. However, to date we did not find successful examples of significant linkages with the private sector.

There is an increasing emphasis on entrepreneurship within the university. The Faculty of Commerce and Management has established the University of Dar es Salaam Entrepreneurship Centre, which has been promoting awareness around entrepreneurship at both the faculty and student levels. It also plays a strong role in incubating small businesses, and has developed a business plan competition with support from the United Nations Development Program.

Another example of entrepreneurial leadership is the University of Dar es Salaam’s College of Engineering and Technology, which has developed a “Clubs, Clusters and Incubators” program with support from the Gatsby Foundation, the Swedish International Development Agency and the World Bank. The program aims at promoting growth of small and medium enterprises in Tanzania and has links with universities in Uganda and Mozambique [15]. Currently they have 8 cluster initiatives focused on technology-specific areas such as vegetable and fruit processing, nutraceuticals, sisal, metal works, cultural tourism, seed, seaweed and mushroom, but none to date has focused on health and biotechnology. The three main activities each cluster initially undertakes includes (1) mobilization of people and resources and analysis of activities, (2) preparation and facilitation of implementation of short term activities, and (3) identification and facilitation of implementation of long-term strategic activities. The success of the pilot clusters has resulted in plans to establish an additional 11 clusters in various areas, including health, across Tanzania. There are transferable lessons to be learnt from this program for other universities and research groups, with respect to knowledge translation and commercialization of local health technologies; for example, how to effectively develop linkages among stakeholders around the focused commercialization of locally relevant technologies, and increasing the capabilities of local firms.

#### Muhimbili University of Health and Applied Sciences (MUHAS)

MUHAS is the country’s premier public medical university, spinning out in 2007 from the UDSM. MUHAS has developed not only as the key medical training institution, but also a strong medical research center, with strong expertise in traditional medicine, clinical trials and diagnostics, and with strong overseas partnerships.

For example, in the 1990s Professor Zul Premji, a researcher at MUHAS, played a critical role in the development of the malaria rapid diagnostic test and its validation along with the company Beckton Dickinson. This rapid diagnostic test is now widely used and available as a product produced by various local and international companies. As Prof. Premji explains “I was the first one to test the validity, sensitivity and that sort of thing of this [rapid diagnostic] test, and then we improved on it quite a bit. Now this test…is commercially available, [and] the prices have gone down because the volumes have increased; now you can get a test for [less than] a dollar.” Beckton Dickinson eventually patented and commercialized the test, but it is unclear if Prof. Premji or Tanzania captured any of the economic value out of its research investment in this area. As Prof. Premji points out “In the early 90s we were still very socialistic. So we didn't know much about these patency rights and all that.” Since then the university has set up a technology transfer office to capture the value of in-house innovation, though to date there appear to have been no success stories.

Professor Fred Mhalu is another pioneering researcher at MUHAS. His research also focuses on adapting point of care diagnostics to local settings for various infectious diseases such as malaria, HIV/ AIDS, and tuberculosis. He also recently completed a phase I study of a multi-gene, multi-clade HIV DNA candidate vaccine in Tanzania as part of a long-standing Swedish-Tanzanian collaboration. At the time of the study he was recruiting for Phase II trials for the HIV vaccine. The vaccine being trialed is manufactured in Sweden and the US and takes into account local conditions, including the types of HIV prevalent.

MUHAS houses the Institute of Traditional Medicine, which plays an important role in traditional medicine research in the country. The institute has a herbarium with an inventory of over 3000 plants being used locally in traditional medicine. It has a total of 22 staff, including 11 academic members. It has developed reasonable capacity in the areas of natural products chemistry and botany but still needs to develop capacity in the area of standardization, biological testing and preclinical drug research. It has been working in the areas of diabetes, benign prostatic hypertrophy, and identification of anti- microbial agents. It has a pilot manufacturing plant which has been producing herbal preparations including mixtures for peptic ulcers, cough, asthma and cholesterol since 2003. The Institute was reportedly also the only research institute that has tried to develop linkages with the private sector, going as far as signing an MoU with a local pharmaceutical manufacturer; to date, the partnership has not translated to concrete advances.

#### Sokoine University of Agriculture

SUA was established in 1984 with a primary focus on agriculture and veterinary sciences. It has around 2600 undergraduate students and 400 graduate students, including about 30 PhD students. The University offers an undergraduate degree in Biotechnology and Laboratory Sciences which has a research-intensive component to it. Currently, the University has about 175 researchers trained at the PhD level in all fields of agriculture, forestry and veterinary medicine. They are establishing a Centre for Biotechnology, in collaboration with the International Centre for Genetic Engineering and Biotechnology (Trieste, Italy), which will have a major focus on agricultural biotechnology. There is significant research infrastructure geared towards agricultural biotechnology research including a recently developed Genomic Sciences and Bioinformatics Centre. The University has also recently established a technology transfer office to stimulate research translation.

#### Ifakara Health Institute

Ifakara Health Institute (IHI) is an autonomous private, non-for-profit organization established with support from the Swiss Tropical Institute. It is best known for its malaria research, but has also expanded to other areas including TB, HIV and other communicable diseases. Examples of research projects it has worked on include the evaluation of the SPf66 vaccine for malaria control, evaluation of insecticide treated bed nets and evaluation of iron supplements to prevent severe anaemia and malaria in infants. It uses an impact-based approach to its research, and played an instrumental role in demonstrating chloroquine resistance in East Africa by mapping and following mutation levels around the country, thus contributing to information showing that the drug was no longer effective.

There have been attempts at product development. For example, the Institute was attempting to develop a molluscoside from a local traditional medicine to act against schistosomiasis-carrying snails. But the product was not clinically efficacious, resulting in the termination of the project. Dr Mshinda, former Director of IHI and current Director General of COSTECH, says that the key barrier to health biotechnology development and translation is around human resources and capital: “I think we need to create an enabling environment for production – first to invest in human resources, and [then] creating an environment which would be more conducive for the private sector to start to invest. So you need human resources, and you need to have also financial and human resource capital. Because the market is there – it's a question of seeing how one can harness it.”

#### Private universities

Private higher educational institutions also play a role in health biotechnology R&D, providing training through their undergraduate and graduate programs for future scientists and technologists as well as doing research. Tumaini University along with its Kilimanjaro Christian Medical College (KCMC) and the Mikocheni International University are key private universities in the health field. KCMC has developed capabilities in health biotechnology and clinical trials. They have a biotechnology laboratory in collaboration with NIMR, and a specialized Clinical Research Centre for TB clinical trials as part of the APRIORI program. The APRIORI program was established at KCMC with support from the Netherlands Organization for Scientific Research, to conduct vaccine trials and drug trials and to develop at the same time a community-focused integral approach for prevention and treatment. KCMC – together with Duke University Medical Center and Kiwakkuki, a community organization fighting HIV/AIDS – receives NIH funding to conduct clinical research on HIV/AIDS, malaria, tuberculosis and other infectious diseases.

Table 1 shows the variety of health-related products and processes that are being developed within Tanzanian research institutions.

**Table 1:** Products and processes being developed in Tanzanian institutions

Product	Health Area	Organization	Description
Artemisinin extraction Process	Malaria	NIMR	Developed an extraction technology that provides greater yield and that has been validated. (No large scale pilot testing yet.)
Herbal Formulation	HIV/AIDS	Institute for Traditional Medicine, Muhimbili University of Health and Applied Sciences	Herbal alkaloid- trepenoid product made from various plants. Proposed to undergo clinical trials.
Herbal Formulation	Benign prostatic hypertrophy	Institute for Traditional Medicine, Muhimbili University of Health and Applied Sciences	Innovative plant based product. Small sample size, placebo-controlled, double-blind clinical trial performed: negative results
Long Lasting Insecticidal Nets	Malaria	A to Z Textiles	Product on market
Antimalarial Vaccines	Malaria	African Malaria Network Trust	Conducting clinical trials at various locations in Tanzania
Acridine Orange based Malaria Diagnostic	Malaria	Muhimbili University of Health and Applied Sciences	Being developed in collaboration with a Japanese organization. Testing a new form of staining for diagnosing malaria.
Chlorproguanil-dapsone-artesunate and other malaria therapies	Malaria	Muhimbili University of Health and Applied Sciences	Running clinical trials for various artesunate combination therapies
Fungal vector control agent	Malaria	Ifakara Health Institute	Testing a fungus with anti – anopheles activity, in collaboration with Imperial College U.K. Fungus is applied into cloth and placed indoors. Doing trial to replicate, look at effectiveness, and evaluate whether production could go to scale

### Private sector

The local pharmaceutical industry in Tanzania appears still in its nascent stages with an estimated local market value in 2007 of around US$33 million [12,16]. Additionally, there is a large market for informal traditional medicines, which are utilized by an estimated 70% of the population. Tanzania’s pharmaceutical industry is serviced by local generic manufacturers, generic manufacturers operating globally (mainly from India and China) and a few large multinational corporations. The local manufacturers service approximately 30% of the country’s market with the rest serviced by importation. Of the local generic manufacturers Shelys Pharmaceuticals and Tanzania Pharmaceutical Industries Ltd (TPI) are the largest players, commanding 50% and 30% of the local manufactured market share respectively. Other manufacturers include Zenufa, Keko Pharmaceutical Industries, Interchem Pharmaceuticals and Mansoor Daya.

Shelys (Dar es Salaam) has over 100 products on the market. TPI (Arusha) was a state-owned pharmaceutical company that was privatized in 1997, and is now owned by a group of private Tanzanian investors and the Tanzanian Investment Funds (with a reported 40:60 split). Both of these firms have made significant South-South partnerships in recent years. In 2008, Aspen Pharmacare of South Africa acquired a 60% stake in Shelys and its linked organization Beta Healthcare International in Kenya. TPI has recently started production of fixed dose antiretroviral combination therapy with technological assistance from Thailand, including technology transfer around manufacturing ARVs from the Faculty of Oriental Medicine at the University of Rangsit in Thailand. Another pharmaceutical company, Zenufa, has recently set up a new manufacturing unit and is in the process of approvals to develop therapeutics such as anti-histamines, amongst other products. Zenufa, Shelys and TPI are all at various stages of setting up and upgrading their manufacturing units to WHO- Good Manufacturing Practices standards that would allow prequalification. Currently Tanzania has no active pharmaceutical ingredient manufacturer. There are numerous industry associations that play a role in advocacy and awareness, including the Tanzania Private Sector Foundation and Tanzanian Association of Pharmaceutical Industry. The key buyers for pharmaceutical products in Tanzania are the government and donor agencies.

A noteworthy example of health product innovation outside of pharmaceuticals is that of A to Z Textile Mills (Arusha). A to Z Textile Mills, in partnership with the Japanese company Sumitomo, has become the largest manufacturer of long-lasting insecticide impregnated bed-nets in Africa. Pellets containing insecticide are shipped from Japan to Arusha, where they are melted, turned into long strings which are rolled onto spools and then formed into nets, cut, packaged and shipped using company owned trucks to points of distribution in many African countries, particularly in East and Central Africa. A to Z currently manufactures about 30 million bed-nets a year, which are WHO-certified and reasonably priced. Moreover, A-Z has created over 5000 jobs for Tanzanians. (A detailed look at A to Z’s strategy and challenges can be found in the A to Z paper in this BMC series).

Our interviews did not find any examples where the local private sector, including pharmaceutical manufacturers, have linkages with the Tanzanian research community in the area of health and biotechnology. This was also the case with A to Z, which only considered foreign technologies when they were initially scoping bednet technologies. During the same time period, Tanzanian researchers were studying and publishing extensively on the malaria parasite and identifying alternative anti-malarial compounds which may have offered alternatives to insecticides used in the bednet.

In our study we found that a lack of shared values between research and private enterprise, low entrepreneurial spirit, and lack of capital are key barriers to collaboration between business and science. Access to capital was reported as a major barrier for both small and medium enterprises and large enterprises, especially with the high interest rates prevalent locally. There is little access to risk capital, local or international, for innovation in general, let alone health and biotechnology. There has been a recent rise in Tanzanian business competitions such as the ones run by the University of Dar es Salaam’s Entrepreneurship Centre, and the “Believe Begin Become" national business plan competition in collaboration with Google and Technoserve. While these are examples of incentivizing entrepreneurship, to date none has been in the medical innovation area. In the 2008 Global Competitiveness Report, businesses ranked infrastructure and an inadequately educated workforce as most problematic factors for doing business in Tanzania.

### NGOs and donors

Donors provide over 70% of funds for health research in Tanzania [17]. The Swedish International Development Agency’s Department for Research Cooperation is one of Tanzania’s chief donors supporting science, especially in health. Swedish support has been concentrated at the University of Dar es Salaam and Muhimbili University College of Health Sciences, the two key institutions involved in research and research training at the time of initiation of the cooperation. More recently it has also taken on funding COSTECH to undergo an organizational review. It supports research project cooperation, faculty support (core support), Information Communication Technology (ICT) infrastructure support, and national access to electronic journals coordinated by the UDSM university library. It also funds regional research programs especially in the field of technology, and activities related to knowledge dissemination.

Other agencies such as the United States Agency for International Development, Norwegian Agency for Development Cooperation, the German development agency GTZ, and the Netherlands Organization for International Cooperation in Higher Education have been less active in funding health research. However, external foundations are increasingly active in funding health research in Tanzania. From 2006 to 2008 foundations such as the Bill & Melinda Gates Foundation, Comic Relief, the Rockefeller Foundation, and the Wellcome Trust provided at least US$16.7 million in direct funding to Tanzanian institutions for health research, with another US$24.8 million in indirect funding.

Given the heavy reliance on donor funding for health research, local researchers were said to focus on areas outlined by the various agencies, whose agendas are cyclical. This results in researchers constantly changing research interests or focus to align with available funding. As Prof. A.E. Pereka, Deputy Vice-Chancellor of Sokoine University of Agriculture points out: "Scientists' research is skewed to donor interest; very often we are caught up in a scenario where we are told this type of research is not very relevant to Africa, because they want to fund projects which they think make a change".

Tanzania has numerous NGOs working in the area of health research and capacity building, such as the African Medical and Research Foundation, a multi- country African NGO focused on health research; and the African Malaria Network Trust (AMANET), which promotes capacity strengthening and networking of malaria R&D in Africa. This latter organization is led by the former Director General of NIMR, Dr Wen Kilama, who points out the importance of African-focused and African-led research: “If you think malaria research is expensive, try malaria”. The network supports various activities including training, capacity strengthening for vaccine clinical trials, and awareness of African malaria research. AMANET is currently in the process of product development of three vaccine candidates for malaria: MSP 3LSP (Merozoite Surface Protein / Long Synthetic peptide), AMA1 (Apical Membrane Antigen) and GMZ2 (Recombinant hybrid of Glutamate Rich protein and MSP 3); it is conducting research and clinical trials across Africa, including in Tanzania. It also conducts workshops in Good Clinical Practice and provides financial support to build local research capacity

## Conclusions

### Strengths and good practices

Tanzania has set up a relatively extensive S&T system with its numerous institution and organizations, both public and private, dedicated to various aspects of sciences and health. There is strong political will towards harnessing local science innovation to address local socioeconomic needs. This is seen through the establishment of a newly dedicated Ministry of Science, Technology and Communication, recent STI policy reform, and the announcement of an increase in governmental funding for scientific R&D from 0.18% to 1% of its GDP, six years ahead of schedule. The reform may provide a policy framework to bring together elements of the existing science and technology innovation (STI) system, and the policy impetus to push towards innovation and commercialization of health research that has so far been missing. Tanzania’s food and drugs regulatory system is a leader in the region, showing leadership in stimulating a strong local pharmaceutical manufacturing industry. We also found that Tanzania has a strong research base in the areas of traditional medicines and diagnostics, increasing capacity in clinical trial research across various institutes such as KCMC, MUHAS, and NIMR.

An exemplar of a model that facilitates commercialization of innovation is the College of Engineering and Technology’s “Clubs, Clusters and Incubators Program” at UDSM. The college spearheaded this initiative of establishing innovation systems and clusters to fast track socioeconomic development in the East African region by harnessing local technologies and entrepreneurship. This program has been successful in terms of developing and translating locally relevant technologies, as well as training for entrepreneurs and establishing linkages between public and private sector partners. The cluster initiatives also provide an effective mechanism to promote innovation and firm competitiveness in target technological areas of local importance such as nutraceuticals, along with opportunities for joint production, research, lobbying and upgrading human resources. The expansion from its original 8 clusters in Tanzania to 19, along with 7 others in collaboration with Makerere University in Uganda and some in Mozambique, attest to its relevance to African countries more widely.

### Recommendations

Tanzania’s health innovation landscape has a large number of institution and organizations both public and private, dedicated to various aspects of sciences and health. In order to have a coherent vision for innovation for development in general and for health in particular, Tanzania may wish to address some key issues.

Like most in the region, universities and research institutes in Tanzania continue to face infrastructure restrictions including access to basic amenities such as water and electricity; and these same infrastructural challenges result in increased manufacturing costs for the local private sector. There is also a lack of investment in scientific infrastructure and its maintenance. Lack of technological capabilities in biological testing, preclinical testing, formulation and standardization, and related areas make it difficult to move from basic research to applications. An analysis of the viability of scaling up local pharmaceutical production has found similar issues [12].

There is a lack of early stage proof of concept funding and venture capital, which are essential to jump start health and biotechnology product development. Nor are there incentives for investments in R&D and its translation for the private sector.

Lack of coordination in research and its funding is also a significant barrier to harnessing local health innovation. All of those interviewed for this study identified missing linkages between the science, business and capital providers as a significant barrier to knowledge flow and translation between actors in the innovation system. COSTECH, which is mandated with the implementation of science policies along with research coordination and its promotion, has been unable to fulfill all these roles due to budgetary and infrastructure constraints. Despite its vision to capture Tanzania’s innovation into socio-economic benefits and poverty alleviation, COSTECH has much ground to cover in terms of establishing linkages with private sector and harnessing the country’s research potential and biodiversity. This was highlighted above in the case of value-added development of Tanzania’s artemisinin locally. However, this seems to be slowly changing; since our interviews, there has been a change in management at COSTECH which has resulted in it undergoing organizational reforms.

Numerous promising technologies have languished on laboratory shelves without seeing the light of day. As Dr Mwele Malachela, Director of Research and Coordination at NIMR points out “I think a lot of the gaps are not real gaps but more about getting the linkages to work. For me, a kind of center would be a good place to have so these ideas are processed, so the visions of all the stakeholders are taken into consideration. So I would look at it from the point of view of the farmer, researcher, down the line to the product, and then full commercialization with private sector – [a] bringing together of the ideas”. The scientific reform undertaken by the ministry may begin to address this lack of linkages.

In his key note address at a consultative workshop on research capacity in Tanzania, the Hon. Dr Ali Mohamed Shein, Vice President of the United Republic of Tanzania, underlined that “The objective of research is to find better ways of solving problems which people face. It is, therefore, imperative that research findings should reach end users, and in this case it is the people” [18]. With an extensive science and technology system, political will including the on going reform of its STI system, and increase in R&D funds, Tanzania seems well positioned for health research advances. Below are recommendations which may help speed that process.

*Recommendation 1: Consolidate groups and policies:* With its diverse S&T framework and numerous policies, the government may wish to consolidate and synergize groups and policies. For example, the Ministry of Health and the Ministry of Communication, Science and Technology have extensive overlap in the coordination of the health research agenda that could all be consolidated under one ministry or agency like COSTECH.

*Recommendation 2: Facilitate South-South collaboration and diaspora linkages:.* There already appear to be partnerships between pharmaceutical firms in Tanzania and those in India, China and Thailand. The governemnt should provide greater mechanisms, funding and channels to expand private sector partnership beyond manufacturing to include innovation. Additionally it should assist in establishing research partnership between its extensive S&T research network and other southern economies. Talented diaspora scientists and businesspeople are also a resource to be tapped.

*Recommendation 3: Increase investments in health and biotechnology product development:* Some of the recent increase in scientific funding could support and encourage proof of concept translational research. Providing incentives to invest in R&D based businesses could include tax breaks and credits for developing in house R&D, stimulation of collaboration between science and business, better infrastructure, easier access to raw material and equipment, and improved distribution networks. Donor agencies should consider moving towards a model of funding that results in longer commitment to health research areas, in combination with knowledge translation and commercialization activities.

*Recommendation 4: Create a mechanism to enhance knowledge translation and linkages between business, science and capital providers*. Establishing an appropriate innovation platform mechanism could stimulate commercialization of Tanzanian health innovation. Such platforms can tap Tanzania’s scientific potential in traditional medicine, diagnostics and clinical research by systematically evaluating the potential of existing R&D through technology scouting and audits nationally.

Since the completion of our case study, we have continued to work with the Ministry of Communication, Science and Technology to address some of the identified key challenges to commercialization of local health research. We jointly hosted a national life sciences workshop in December 2007 in Dar es Salaam where we presented the case study results and recommendations, which were discussed with local stakeholders, including government officials, people working in the private sector, and members of the research community. Stakeholders supported our results and recommendations especially the need to increase knowledge flow. A local steering committee has been formed for strategy and planning to develop a life sciences innovation center [19,20].

It is our hope that, through deeper understanding of Tanzania’s current capabilities and future options, the country can better realize its potential for science-based health innovation.

## Competing interests

None declared.

## Authors' contributions

RS, PAS, ASD contributed to the concept and design of this study and participated in site visits, analyzed the findings, and participated in manuscript development.
